# Lithium-Associated Hypothyroidism in a Specialized Lithium Clinic at a Tertiary Care Hospital in Riyadh, Saudi Arabia: A Retrospective Study

**DOI:** 10.3390/healthcare12060636

**Published:** 2024-03-12

**Authors:** Ahmad H. Almadani, Maha S. Algazlan, Abdulaziz F. Alfraiji, Nawaf Y. Almalki

**Affiliations:** 1 Department of Psychiatry, College of Medicine, King Saud University, Riyadh 11451, Saudi Arabia; 2 Department of Psychiatry, King Saud University Medical City, King Saud University, Riyadh 11362, Saudi Arabia; nyalmalki@gmail.com (N.Y.A.); 3 SABIC Psychological Health Research and Applications Chair (SPHRAC), Department of Psychiatry, College of Medicine, King Saud University, Riyadh 12372, Saudi Arabia

**Keywords:** lithium, hypothyroidism, psychotropic medications, Saudi Arabia, mental illness

## Abstract

Hypothyroidism is a common side effect of lithium use and is associated with a slower response to treatment and poorer long-term remission in patients with bipolar disorder. No studies have examined the prevalence of lithium-associated hypothyroidism or its associated factors in Saudi Arabia. We aimed to estimate the prevalence of lithium-associated hypothyroidism among psychiatric patients in a specialized lithium clinic at a tertiary care hospital in Saudi Arabia and to examine the possible risk factors for its development. This retrospective observational study included 117 participants. The prevalence of secondary hypothyroidism was 15%. The median duration between the start of lithium therapy and the first abnormal thyroid test result was 341 days. The multiple logistic regression showed that none of the studied variables, namely, sex, current age, age of lithium initiation, continuity on lithium, lithium level (≤0.5 mmol/L or >0.5 mmol/L), and prescription of as-needed medications, was significantly associated with secondary hypothyroidism. Our study also shed light on the possible clinical significance of baseline TSH levels in developing hypothyroidism secondary to lithium. Further multicenter studies with larger sample sizes are warranted to examine the generalizability of these results.

## 1. Introduction

Lithium, a mood stabilizer, is considered an effective medication in psychiatry [1]. This medication was first used in 1949 by John Frederick Joseph Cade, an Australian psychiatrist, to treat manic episodes in patients with bipolar disorders (BDs) [2]. In addition, lithium was used to manage recurrent depressive disorders in the 19th century by a Danish physician, Eric Lange [3]. Lithium is still one of the most established long-term pharmacotherapies for BDs, preventing both depressive and manic episodes as well as reducing suicide risk independently of its mood-stabilizing effect [4].

Lithium carbonate is orally administered and is easily absorbed from the gastrointestinal tract [5]. It is excreted by the kidneys (95–98%); hence, its concentration in the blood is significantly affected by the condition of the patient’s kidneys, sodium intake, and age [5]. Lithium can enter cellular and extracellular fluids, as well as breast milk. Its therapeutic concentration ranges from 0.4 to 1.2 mmol/L, depending on the stage of illness, among other factors [5,6]. The antimanic, antidepressant, and prophylactic stabilizing effects of lithium, in addition to the complexity of BD, suggest its ability to act at multiple sites [7]. Lithium also modulates the expression of genes involved in the regulation of degenerative and regenerative processes, which contributes to its neuroprotective effects [7].

However, lithium has been associated with gastrointestinal, neuromuscular, and endocrine adverse effects in 35–93% of patients receiving it [8]. With regard to endocrine side effects, thyroid gland disorders, including hypothyroidism and goiter, are the most common [9]. Most lithium-associated hypothyroidism cases are subclinical [10], identified by an elevated thyroid-stimulating hormone (TSH) level and normal serum-free thyroxine (FT4) concentration, with few or no clinical signs or symptoms of thyroid dysfunction [11]. Lithium accumulates in the thyroid gland at concentrations three to four-fold greater than in plasma [12] and has an antithyroid effect that involves several mechanisms. It also reduces the production of thyroid hormones and inhibits their release into the body. Moreover, lithium can alter the immune processes in the thyroid gland [13].

Clinical hypothyroidism affects several cognitive domains. A review addressed deficits in general intelligence, attention and concentration, memory, perceptual function, language, psychomotor function, and executive function, of which memory is the domain most consistently affected [14]. Subclinical hypothyroidism exhibits many psychiatric manifestations, such as cognitive dysfunction, mood disturbances, and decreased responses to standard psychiatric treatments [15].

The treatment setting in which lithium is prescribed (i.e., strict clinical settings such as a specialized lithium clinic versus less strict naturalistic settings in which lithium is prescribed by general practitioners or general psychiatrists in non-specialized lithium clinics) is considered a major factor influencing the treatment mortality rate, in addition to the usual side effects of lithium [16]. Similarly, lithium administration in specialized lithium clinics decreases the mortality rate of patients with affective illnesses [17]. Changes in the setting in which the drug is administered from a strict regime to a naturalistic setting do not affect the efficacy of the drug. However, the continued properties of a drug given in a naturalistic setting are dependent on the characteristics of the setting, such as avoidance of termination or frequent discontinuations [16].

Studies have explored the prevalence of thyroid dysfunction, particularly hypothyroidism, among patients with psychiatric disorders receiving lithium. Lithium-induced hypothyroidism is highly prevalent among psychiatric patients on lithium and is positively correlated with age, female sex, duration of lithium use, and family history of thyroid disturbances [18,19,20,21,22].

Hypothyroidism is one of the most common side effects of lithium [9] and is associated with a slower response to acute treatment and poorer long-term remission in patients with BD-I [20]. To the best of our knowledge, no previous study has examined the prevalence of lithium-associated hypothyroidism in Saudi Arabia. We, therefore, aimed to estimate the prevalence of lithium-associated hypothyroidism among psychiatric patients in a specialized lithium clinic at a tertiary care hospital in Riyadh, Saudi Arabia, and to examine the factors associated with its development. We hypothesized that lithium intake contributes to the development of hypothyroidism and that lithium-associated hypothyroidism is highly prevalent among psychiatric patients at lithium clinics. Moreover, as suggested previously [18,19,20,21,22], we hypothesize that positive correlations between lithium-associated hypothyroidism and specific factors are likely to be found. Owing to the scarcity of studies in the region, this study adds value to the literature by assessing this under-studied population with an optimistic explorative nature toward the future of lithium initiation and monitoring guidelines.

## 2. Material and Methods

### 2.1. Design and Participants

This was a retrospective observational cross-sectional study conducted at the Lithium Clinic, a specialized psychiatric clinic in the Adult Unit of the Department of Psychiatry at King Saud University Medical City (KSUMC), Riyadh, Saudi Arabia, which is a university-affiliated tertiary government hospital. This specialized lithium clinic was established to follow up on patients who were prescribed lithium, regardless of their psychiatric diagnoses. The study’s target population consisted of patients followed up by the Lithium Clinic since its establishment in late 2018. All patients followed up in the Lithium Clinic who were prescribed lithium at least once, as shown in their electronic chart documentation, were included and those who were not shown to have been prescribed lithium were excluded from the study. Ethical approval for the study and permission to access patient medical charts were obtained from the Institutional Review Board of the College of Medicine at King Saud University, Riyadh, Saudi Arabia (Research Project No. E-21-6416).

### 2.2. Data Collection and Sample Size

Data were collected from the electronic health records of KSUMC for Integrated Health Information (eSIHI), the internal name of the Cerner Millennium, from January to March 2022. During this period, the research team conducted manual data collection, starting with the first clinic appointment list available in the system (December 2019) until January 2022, and accessed the files and collected the information. However, the electronic appointment booking system did not provide any appointments before December 2019. A total of 117 participants were included in this study.

### 2.3. Variables

Concerning the diagnosis of BD, in some participant charts, the subtype of the disease (i.e., BD I or II) was not specified; therefore, we opted to combine BD I and II into a single category, namely bipolar and related disorders. Furthermore, we added two additional groups aiming for simple diagnostic categorization, namely (1) bipolar and related disorders with comorbidities and (2) others. As such, there were three main groups, namely (1) bipolar and related disorders; (2) bipolar and related disorders with comorbidities; and (3) others. All the diagnoses were based on the latest clinical notes in the charts.

We collected lithium-related information, including (1) age at initiation of lithium treatment; (2) continuity of lithium according to the most recently written documentation; (3) reasons for lithium discontinuation (if applicable); (4) side effects leading to discontinuation; and (5) lithium level collected at three intervals: (a) the earliest documented lithium level, (b) lithium level after an interval of one year, and (c) the most recently documented level.

We recorded thyroid-related information such as (1) the presence of hypothyroidism (based on a documented diagnosis); (2) age at onset of thyroid illness; (3) thyroid illness development (prior or post-lithium prescription); (4) duration of lithium treatment until the first abnormal thyroid function results; (5) whether the patient was assessed by other medical services for thyroid-related illnesses/abnormalities; (6) levothyroxine status (whether or not patient was taking levothyroxine); and (7) thyroid function tests (TSH and T4) collected in the following intervals depending on the following available results: documented baseline, first documented test available on lithium, documented from 3 months up to 3 years after the first available test on lithium, most recent test on lithium, and post-lithium if discontinued.

Finally, we collected information regarding medication, including regularly prescribed psychotropics (monotherapy, polytherapy, or no medication). Medications were categorized into antipsychotics, antidepressants, benzodiazepines, and antiepileptics. Other information collected included the generic names of the medications co-prescribed with lithium and the prescriptions of medications, as needed. As-needed medications were categorized as antipsychotics, benzodiazepines, or others.

### 2.4. Statistical Analysis

Descriptive statistics of the data are presented with *n* (%) for categorical variables, non-normalized variables (for non-parametric tests) are shown as median (Q1 and Q3), and normalized variables (for parametric tests) are shown as mean ± SD. The mean TSH level was calculated based on the first available TSH level on lithium, the TSH level 3 months to 3 years after the first available TSH level on lithium, and the most recent TSH level on lithium. The mean T4 level was calculated based on the first T4 level available on lithium, the T4 level over 3 months to 3 years after the first T4 level available on lithium, and the most recent T4 level on lithium. The mean lithium level was calculated based on the earliest documented lithium level, the lithium level approximately one year after the earliest documented level, and the most recently documented lithium level. The association between different hypothyroidism groups and demographic and lithium-related factors was studied using the chi-square test. Bonferroni correction was used for post-hoc analysis (the same alpha level of 0.05), as the *p*-value was adjusted by multiplying the resulting *p*-value of the pairwise comparison by the number of comparisons. This is integrated within the software used and yields the same result as adjusting the alpha level by dividing it by the number of comparisons). The association between the continuity of lithium use and demographic and lithium-related factors was also studied using the chi-squared test. The Fisher–Freeman–Halton exact test was used when the assumption of at least 80% of the cells having an expected count of five or more for the chi-square test occurred. A comparison of the current age and age at lithium initiation was made using a one-way analysis of variance and an independent t-test with Bonferroni correction was used for post-hoc analysis (the same alpha level of 0.05), as the *p*-value was adjusted. Multiple logistic regression was used to study the variables, namely, sex, current age, age of lithium initiation, continuity on lithium, lithium level (≤ 0.5 mmol/L or >0.5 mmol/L), and prescription of as-needed medications association with having secondary hypothyroidism. The correlation between different Barnard levels of thyroid function tests and lithium levels was studied using Pearson’s correlation coefficient or Spearman’s correlation coefficient [23]. The normality of data distribution was tested using the Shapiro–Wilk test. IBM SPSS 28 for Windows (IBM Corp., Armonk, NY, USA) was used for statistical analysis and *p*-values < 0.05 were considered statistically significant.

## 3. Results

As shown in Table 1, a sample of 117 participants were included in the study, of whom 92 (79%) were females. Most of the study participants were between 31 and 49 years of age (48 [41%]) and 44 (38%) were 30 years of age or younger. The mean age of the study group was 37.51 ± 11.86 years and the median (Q1 and Q3) was 35 (27 and 48). More than two-thirds (83 [71%]) of the participants had been diagnosed with bipolar and related disorders and only 10 (9%) had bipolar and related disorders with comorbidities. Among the “others” diagnostic category (24 [20%]), 10 participants (42%) were diagnosed with major depressive disorder and 9 (38%) had been diagnosed with schizoaffective disorder.

As shown in Table 2, 46 (39%) of participants initiated lithium intake at an age ≤ 30 years. The mean patient age at lithium initiation was 20.49 ± 11.40 years and the median (Q1 and Q3) was 18 (10 and 34). More than two-thirds (84 [72%]) of the participants were still on lithium at the time of the study. Among 33 participants who discontinued lithium intake, 12 (36%) discontinued lithium due to side effects. The most frequent side effect was lethargy, which five (42%) participants encountered.

As shown in Table 3, 37 (32%) of the participants had hypothyroidism. The prevalence of secondary hypothyroidism was 15% (14 cases had hypothyroidism after lithium intake). The median (Q1 and Q3) time between the start of lithium therapy and the first abnormal thyroid test result was 341 days (98 and 601). The median (Q1 and Q3) age at the onset of thyroid illness was 29 years (25 and 39). Of the participants, 35 (95%) out of 37 who had hypothyroidism were on levothyroxine medications.

As shown in Table 4, 90 (77%) of the participants were on polytherapy and 67 (57%) were on lithium and others. The most frequently prescribed medication group was antipsychotics (which were prescribed to 83 [92%] of the participants), then antidepressants (30 [33%] participants), antiepileptics (20 [22%] participants), benzodiazepines (6 [7%] participants), and others (prescribed to 6 [7%] participants). Most of the participants, specifically 103 (88%), did not take pro re nata (PRN) medication and only 14 (12%) took PRN medication. Antipsychotic medication was the most frequently prescribed PRN medication (10 [71%] participants).

Chi-square or exact tests were conducted, as shown in Table 5, to compare hypothyroidism status and demographic and lithium-related factors. There was a statistically significant association between sex and hypothyroidism status (*p* = 0.012): 57 (67%) of females had no hypothyroidism, 13 (15%) had secondary hypothyroidism, and 15 (18%) had primary hypothyroidism. In comparison, 23 (96%) of males had no hypothyroidism, one (4%) had secondary hypothyroidism, and none (0%) had primary hypothyroidism. The age of initiation of lithium showed a statistically significant difference, as it was lower for the no hypothyroidism group (mean = 28.58 ± 10.00 years) than the primary hypothyroidism group (mean = 36.93 ± 12.68 years) (*p* = 0.034).

As presented in Table 6, multiple logistic regression showed that none of the studied variables had a statistically significant association with secondary hypothyroidism.

Table 7 shows the correlations between different lithium intake states, mean lithium levels, different thyroid function tests, and mean TSH and T4 levels in patients who were not on levothyroxine. There was a moderately positive relationship between the first TSH level available on lithium and the TSH baseline off lithium; Spearman’s correlation coefficient = 0.457, *p* = 0.001. There was a moderately positive relationship between the TSH baseline off lithium and TSH levels over 3 months to 3-year intervals after the first TSH level available on lithium; Spearman’s correlation coefficient = 0.536, *p* = 0.002. Another moderately positive relationship was found between the most recent TSH level on lithium and the TSH baseline off lithium; Spearman’s correlation coefficient = 0.494, *p* < 0.001. There was a moderate-to-strong positive relationship between the most recent TSH level of lithium and the baseline TSH level of lithium; Spearman’s correlation coefficient = 0.625, *p* = 0.013. There was a strong positive relationship between the most recent T4 level of lithium and the T4 baseline of lithium; Pearson’s correlation coefficient = 0.726, *p* = 0.003. There was a weak positive relationship between the mean lithium and TSH levels; Spearman’s correlation coefficient = 0.249, *p* = 0.028. There was a weak negative relationship between mean T4 and mean lithium levels; Spearman’s correlation coefficient = −0.234, *p* = 0.038. As shown in Table 6, there was a moderate positive correlation between the mean TSH level and the baseline TSH off lithium; Spearman’s correlation coefficient = 0.560, *p*-value < 0.001.

## 4. Discussion

The prevalence of lithium-induced hypothyroidism was found to be 15%. This figure is greater than the rate of overt hypothyroidism in the general population, with a mean of 3.05% in Europe [24]. However, our result is comparable to the prevalence among patients using lithium in other studies, that is, 10.3% in the United Kingdom [21] and 9% in Iran [22]. The high prevalence in our study could be explained by the predominance of female participants in our sample. This is the first study to explore the prevalence and risk factors of lithium-associated hypothyroidism in psychiatric patients in Saudi Arabia.

More than half of our participants were diagnosed with bipolar and related disorders without comorbidities and more than half received polytherapy with lithium and another psychotropic medication. These findings, namely the diagnosis and polypharmacy status, could indicate the possible presence of additional contributing factors in the development of hypothyroidism, such as the psychiatric illness itself and the side effects of other psychotropic medications used concomitantly [20]. Previous studies have suggested that patients with BD have a higher propensity to develop thyroid abnormalities [20].

Among the participants in our study, the median period between starting lithium treatment and the first abnormal thyroid result was 341 days. Aligned with our findings, the average duration of lithium use prior to the diagnosis of hypothyroidism was found to be about 18 months in a study conducted by Chakrabarti [25]. In addition, we found that lithium was initiated earlier for euthyroid patients than for patients with primary hypothyroidism. We hypothesize that this finding might reflect the psychiatrists’ reluctance to prescribe lithium for patients who have hypothyroidism. This hypothesis is aligned with another study indicating that psychiatrists were skeptical about starting lithium due to several perceived barriers, among which are the adverse effects [26].

A study in Poland found that females demonstrated greater vulnerability to thyroid dysfunction during lithium treatment [18]; conversely, in our research, we found no significant association between secondary hypothyroidism and sex. However, our findings concerning sex are in line with a study conducted in Iran [19], in which sex was found to have no significant association with the development of subclinical hypothyroidism after lithium use. We hypothesize that our findings might be explained by the differing population compared to Western countries as opposed to the study conducted in Iran, which is considered part of the Middle East. Alternatively, our findings might be explained by the small number of secondary hypothyroidism in our sample when compared to the euthyroid group. Regarding age and secondary hypothyroidism, our results revealed no significant associations between the two. Alternatively, another study found that the risk for hypothyroidism was notably elevated in women who were older than 50 years [27]. Although most of our sample were females, this might be due to our sample distribution, as approximately three-quarters of the patients were younger than 50 years. 

Our results suggest that the higher the baseline TSH level before lithium initiation, the more likely the development of hypothyroidism secondary to lithium, as a positive correlation was found between baseline TSH off lithium and the mean TSH level on lithium. This finding is in line with studies indicating that a high baseline TSH level (prior to the diagnosis of primary hypothyroidism) is a predictor of the development of primary hypothyroidism [28] and postoperative hypothyroidism [29] in the general population.

The reversibility of lithium-associated hypothyroidism after lithium discontinuation has been presented [30]. In our study, there was no significant correlation between the mean TSH on lithium and TSH levels after discontinuation of lithium. We believe that multiple factors contributed to the contradictory findings of our study. First, in our study, only two participants (6%) of those who stopped lithium had a prior diagnosis of secondary hypothyroidism, which limited our ability to detect a significant correlation. Second, in the aforementioned study [30], the clinical indicators that led to the decision to stop thyroid replacement therapy were detected after a mean period of 9.2 ± 10.5 months. However, the nature of our retrospective observational study precluded the presence of a specific time constraint on post-lithium TSH levels.

Regarding lithium levels and hypothyroidism, we found a weak positive relationship between mean lithium and TSH levels and a weak negative relationship between mean T4 and lithium levels. This is consistent with a study conducted by Tellian et al., which suggested that lithium-induced changes in TSH levels are serum level-dependent [31]. This weak relationship could be explained by the limitations of the sample size.

Our study has several strengths. It was the first of its kind conducted in Saudi Arabia and the study was conducted in a highly specialized clinic, the Lithium Clinic, which is considered very rare in Saudi Arabia. The study also sheds light on the use of lithium not only in mood disorders but also in other psychiatric conditions. In comparison with other studies, we examined laboratory indicators of hypothyroidism (TSH and T4 levels) at five different time points (documented baseline, first documented test available on lithium, documented from 3 months to 3 years after the first available test on lithium, most recent test on lithium, and post-lithium if discontinued). This enriched our findings with clinically relevant data related to laboratory risk factors for lithium-induced hypothyroidism. In addition, our results were more comprehensive regarding participant pharmacological management than those of other studies. We have included details regarding the pharmacological status, other medications used (including PRNs), and their possible correlation with lithium-induced hypothyroidism. Finally, our study highlights the need for and the benefit of similar specialized psychiatric clinics in Saudi Arabia as such clinics could potentially ensure a more thorough assessment and regular monitoring of factors related to lithium and hypothyroidism than regular clinics.

Our study also has certain limitations. First, our study design compelled our sampling to rely on a single specialized lithium clinic, which does raise the possibility of selection bias and has forced several factors, namely, a limited sample size given the number of patients in the clinic, a skewed population (more females and younger patients), and a lack of long-term follow-up. Furthermore, our study design lacked control participants to investigate the true effect of lithium on thyroid function. Second, due to documentation-related limitations, we did not evaluate the course and severity of psychiatric illness or the number of hospitalizations. Also, for the same reason, in some patient diagnoses (e.g., bipolar diagnosis), the subtype of the disorder was not specified (e.g., type I or II). Therefore, we performed the stated diagnostic categorization. In addition, due to limited documentation, we did not examine the participants’ socioeconomic variables or family history, which could have yielded other significant correlations. Third, given the nature of our retrospective study, we could not obtain specific laboratory data, such as thyroid autoantibodies, which could enrich our understanding of the autoimmune aspects of this illness. Of note, other studies have compared lithium-induced hypothyroidism to primary hypothyroidism. Unfortunately, we lacked the national registry prevalence rates of primary hypothyroidism for comparison. Finally, we could not access medical charts before December 2019 because of system restrictions.

We recommend that future studies explore other variables not examined in this study, such as socioeconomic variables, family history, specific diagnoses, factors influencing severity (e.g., disease course and number of hospitalizations), and renal function tests. Moreover, considering the current findings suggesting that pre-lithium TSH level is a predictor of the development of secondary hypothyroidism, we recommend that future studies elaborate on this relationship to aid physicians and patients in making informed decisions about lithium initiation. Another future direction that the authors propose includes conducting multicenter studies with larger sample sizes to examine the generalizability of the results on a broader scale.

## 5. Conclusions

Our study is the first to explore the prevalence of secondary hypothyroidism due to lithium in Saudi Arabia, indicating a relatively high prevalence. It also suggests that the higher the baseline TSH level before lithium initiation, the greater the likelihood of developing hypothyroidism secondary to lithium.

## Figures and Tables

**Table 1:** Demographic information and psychiatric diagnosis.

Demographic Information	Value
Sex, *n* (%)	
Female	92 (79%)
Male	25 (21%)
Age, mean ± SD/median (Q1, Q3)	37.51 ± 11.86/35 (27,48)
≤30 Years	44 (38%)
Between 31 to 49 Years	48 (41%)
≥50 Years	25 (21%)
Most recent psychiatric diagnosis	
Bipolar and related disorders	83 (71%)
Bipolar and related disorders + Comorbidity	10 (9%)
Others	24 (20%)
Comorbidities *	
Borderline personality disorder	2 (20%)
Generalized anxiety disorder	1 (10%)
Intellectual disability	1 (10%)
Obsessive compulsive disorder	2 (20%)
Post-traumatic stress disorder	1 (10%)
Substance use disorder	4 (40%)
Gender dysphoria	2 (20%)
Others *	
Major depressive disorder	10 (42%)
Schizoaffective disorder	9 (38%)
Post-traumatic stress disorder	1 (4%)
Obsessive compulsive disorder	1 (4%)
Schizophrenia	3 (12%)
Dependent personality disorder	1 (4%)

* Multiple options are allowed. Data are presented as *n*, (%), unless otherwise noted.

**Table 2:** Lithium-related information.

Characteristics	Value
Lithium age of initiation, mean ± SD median (Q1, Q3)	20.49 ± 11.40/18 (10, 34)
Not documented	37 (32%)
≤30 Years	46 (39%)
From 31 to 49 Years	29 (25%)
≥50 Years	5 (4%)
Continuity on lithium *	
No	33 (28%)
Yes	84 (72%)
Reason for lithium discontinuation (among 33 participants) **	
Side effects	12 (36%)
Compliance and preference	8 (24%)
Response	7 (21%)
Not documented	5 (15%)
Other (suicidal attempts, pregnancy, ECG changes)	5 (15%)
Side effects contributing to lithium discontinuation (among 12 participants) **	
Lethargy	5 (42%)
Weight gain	2 (17%)
Dullness	2 (17%)
Decreased libido	1 (8%)
Acne	1 (8%)
Flank pain	1 (8%)
GI related	1 (8%)
Metallic taste	1 (8%)
Kidney related	1 (8%)
Dry mouth	1 (8%)
Thyroid-related	1 (8%)
Lithium levels	
Earliest documented lithium level, mean ± SD	0.55 ± 0.25
Lithium level approximately 1 year after the earliest documented level, mean ± SD	0.59 ± 0.21
Most recently documented lithium level, mean ± SD	0.63 ± 0.24
Mean lithium level ^§^, mean ± SD	0.59 ± 0.17
≤0.5 mmol/L	38 (32%)
>0.5 mmol/L	79 (68%)

Data are presented as *n*, (%), unless otherwise noted. Lithium levels unit (mmol/L). * Continuity of lithium according to the most recently written documentation. ** Multiple options are allowed. ^§^ Calculated as the mean of the earliest documented lithium level, lithium level approximately one year after the earliest documented level, and the most recently documented lithium level.

**Table 3:** Thyroid-related information.

	*n* (%)/Median (Q1, Q3)
Known hypothyroidism (overall prevalence)	No 80 (68%) Yes 37 (32%)
Prevalence of secondary hypothyroidism (out of 94 patients)	No 80 (85%) Yes 14 (15%)
Hypothyroid before or after lithium	After 14 (48%) Before 15 (52%)
Duration between starting lithium and first abnormal thyroid result in days	341 (98, 601)
Thyroid illness age of onset	29 (25, 39)
Assessment of thyroid status by other services	No 94 (80%) Yes 23 (20%)
Levothyroxine medication	No 82 (70%) Yes 35 (30%)

**Table 4:** Medication-related information.

	*n* (%)
Pharmacological status	
Mono	21 (18%)
Poly ^§^	90 (77%)
None	6 (5%)
Medication groups	
Lithium and others	67 (57%)
Lithium only	17 (15%)
None	6 (5%)
Others	27 (23%)
Regular polytherapy medications: (Antipsychotic, Antidepressant, Benzodiazepines, Anti-epileptic, Others) (among 90 participants) *	
Antipsychotic	83 (92%)
Antidepressant	30 (33%)
Anti-Epileptic	20 (22%)
Benzodiazepines	6 (7%)
Others	6 (7%)
Lithium co-prescribed regular medications (among 67 participants) *	
Quetiapine	6 (7%)
Olanzapine	6 (7%)
Aripiprazole	4 (6%)
Bupropion	4 (6%)
Escitalopram	4 (6%)
Mirtazapine	3 (4%)
Lorazepam	3 (4%)
Procyclidine	3 (4%)
Risperidone	3 (4%)
Risperidone LAI **	4 (6%)
Amisulpride	2 (3%)
Clozapine	2 (3%)
Haloperidol	2 (3%)
Valproic acid	2 (3%)
Duloxetine	1 (1%)
Propranolol	1 (1%)
Clomipramine	1 (1%)
Gabapentin	1 (1%)
Carbamazepine	1 (1%)
Lamotrigine	1 (1%)
Venlafaxine	1 (1%)
Sulpiride	1 (1%)
Fluvoxamine	1 (1%)
Trifluoperazine	1 (1%)
As-needed medication (PRN medication)	
No	103 (88%)
Yes	14 (12%)
Prescribed as-needed medications: (PRN medications groups) (among 14 participants) *	
Antipsychotic	10 (71%)
Benzodiazepines	6 (43%)
Procyclidine	4 (29%)

^§^ Two or more medications. * Multiple options are allowed. ** Long-acting injection.

**Table 5:** Association between hypothyroidism status and demographic and lithium-related factors.

		Hypothyroidism Status			*p*-Value	*p*-Value for Post-Hoc
		No Hypothyroidism	Secondary Hypothyroidism	Primary Hypothyroidism		
Current age	Mean ± SD	35.98 ± 11.41	39.50 ± 10.54	40.20 ± 13.04	0.299	
Age at initiation of Lithium	Mean ± SD	28.58 ± 10.00	31.56 ± 10.57	36.93 ± 12.68	0.034	1.000 ^a^ 0.030 ^b^ 0.714 ^c^
		*n* = 80 (%)	*n* = 14 (%)	*n* = 15 (%)		
Sex	Female	57 (67)	13 (15)	15 (18)	0.012	0.228 ^a^ 0.030 ^b^ 1.000 ^c^
	Male	23 (96)	1 (4)	0 (0.00)		
Continuity (still on Lithium)	No	24 (75)	2 (6)	6 (19)	0.316	
	Yes	56 (73)	12 (15)	9 (12)		
Lithium level	≤0.5 mmol/L	26 (72)	2 (6)	8 (22)	0.086	
	>0.5 mmol/L	54 (74)	12 (16)	7 (10)		
Pharmacological status	Mono	16 (80)	0 (0)	4 (20)	0.208	
	None	5 (83)	0 (0)	1 (17)		
	Poly	59 (71)	14 (17)	10 (12)		
Regular medication groups	Lithium and others	43 (70)	12 (20)	6 (10)	0.279	
	Lithium only	13 (81)	0 (0)	3 (19)		
	None	5 (83)	0 (0)	1 (17)		
	Others	19 (73)	2 (8)	5 (19)		
As-needed medication (PRN medication)	No	70 (74)	10 (10)	15 (16)	0.082	
	Yes	10 (71)	4 (29)	0 (0)		

^a^ No hypothyroidism vs. secondary hypothyroidism. ^b^ No hypothyroidism vs. primary hypothyroidism. ^c^ Secondary hypothyroidism vs. primary hypothyroidism.

**Table 6:** Multiple logistic regression for the factors associated with having secondary hypothyroidism.

	Unadjusted OR	*p*-Value	95% CI of OR		Adjusted OR	*p*-Value	95% CI of OR	
Sex								
Male	1				1			
Female	5.25	0.120	0.65	42.44	7.18	0.076	0.81	63.45
Current Age	1.04	0.041	1.00	1.07	1.05	0.285	0.96	1.14
Age of lithium initiation	1.03	0.144	0.99	1.06	0.99	0.831	0.91	1.08
Continuity (still on Lithium)								
No	1				1			
Yes	2.57	0.239	0.53	12.38	2.05	0.402	0.38	11.07
Lithium level								
≤ 0.5 mmol/L	1				1			
> 0.5 mmol/L	1.00	0.994	0.44	2.31	2.26	0.338	0.43	11.93
As-needed medication (PRN medication)								
No	1				1			
Yes	2.80	0.131	0.74	10.65	2.59	0.214	0.58	11.58

**Table 7:** Correlation between different thyroid function tests according to lithium intake states, mean lithium level, mean TSH, and T4 levels.

Thyroid Function Variables	Correlation Coefficient	*p*-Value
TSH first available on lithium and TSH baseline off lithium	0.457 ^a^	0.001
T4 first available on lithium and T4 baseline off lithium	0.284 ^b^	0.062
TSH 3 months—3-year interval after first available on lithium and TSH baseline off lithium	0.536 ^a^	0.002
T4 3 months—3-year interval after first available on lithium and T4 baseline off lithium	0.284 ^b^	0.062
TSH latest on lithium and TSH baseline off lithium	0.494 ^a^	<0.001
T4 latest on lithium and T4 baseline off lithium	0.092 ^b^	0.552
TSH latest off lithium and TSH baseline off lithium	0.625 ^a^	0.013
T4 Latest off lithium and T4 baseline off lithium	0.726 ^b^	0.003
Mean thyroid and lithium level values	Correlation Coefficient	*p*-value
Mean TSH level * and TSH baseline off lithium	0.560 ^a^	<0.001
Mean T4 level ** and T4 baseline off lithium	0.271 ^b^	0.075
Mean TSH level * and TSH latest off lithium	0.322 ^a^	0.308
Mean T4 level ** and T4 latest off lithium	0.246 ^b^	0.419
Mean lithium level ^§^ and Mean TSH *	0.249 ^a^	0.028
Mean lithium level ^§^ and Mean T4 **	−0.234 ^a^	0.038

^a^ Spearman’s correlation coefficient. ^b^ Pearson’s correlation coefficient. * Mean TSH level is calculated as the mean of TSH first available on lithium, TSH 3 months to 3 years after first available on lithium, and TSH latest available on lithium. ** Mean T4 level is calculated as the mean of T4 first available on lithium, T4 3 months to 3 years after first available on lithium, and T4 latest on lithium. ^§^ Mean lithium level is calculated as the mean of the earliest documented lithium level, approximately 1-year lithium level after earliest documented, and latest documented lithium level. Lithium levels unit (mmol/L).

## Data Availability

Data of this study will be made available upon reasonable request directed to the corresponding author.
